# A population model for the 2017/18 listeriosis outbreak in South Africa

**DOI:** 10.1371/journal.pone.0229901

**Published:** 2020-03-12

**Authors:** Peter Joseph Witbooi, Charlene Africa, Alan Christoffels, Ibrahim Hussin Ibrahim Ahmed

**Affiliations:** 1 Department of Mathematics and Applied Mathematics, University of the Western Cape, Bellville, South Africa; 2 Department of Medical Biosciences, University of the Western Cape, Bellville, South Africa; 3 SA MRC Bioinformatics Unit, South African National Bioinformatics Institute, University of the Western Cape, Bellville, South Africa; Heidelberg Germany, GERMANY

## Abstract

We introduce a compartmental model of ordinary differential equations for the population dynamics of listeriosis, and we derive a model for analysing a listeriosis outbreak. The model explicitly accommodates neonatal infections. Similarly as is common in cholera modeling, we include a compartment to represent the reservoir of bacteria. We also include a compartment to represent the incubation phase. For the 2017/18 listeriosis outbreak that happened in South Africa, we calculate the time pattern and intensity of the force of infection, and we determine numerical values for some of the parameters in the model. The model is calibrated using South African data, together with existing data in the open literature not necessarily from South Africa. We make projections on the future outlook of the epidemiology of the disease and the possibility of eradication.

## Introduction

The World Health Organisation (WHO, 2018) reported [1] the 2017/2018 outbreak of listeriosis in South Africa as the largest global outbreak recorded to date. Between 1 January 2017 and 14 March 2018, the National Institute for Communicable Diseases (NICD) reported 978 confirmed cases of whom 42% were neonates, infected via vertical transmission from infected pregnant mothers. Listeriosis is transmitted through the contamination of infected food products with a Gram-positive non-spore forming, facultatively anaerobic bacillus called *Listeria monocytogenes* [2] (Lamont et al 2011). The outbreak was predominantly attributed to sequence type 6 (ST6) while other sequence types attributed to 9% of infections [1] (WHO, 2018). Listeriosis is reported to occur 18 times more frequently in pregnant than non-pregnant women [3] (Jackson et al., 2010). Infection is usually asymptomatic or may primarily manifest as a non-specific flu-like illness which is often overlooked, particularly during pregnancy, leaving the mother and her unborn infant at risk of serious illness. The infection may be vertically transmitted while the baby is in utero, either by translocation across the placenta or by aspiration of contaminated amniotic fluid or or during delivery of the infant. Foetal infection (during the 1st and 2nd trimester of pregnancy) may result in abortion, stillbirth, or premature delivery with amnionitis (during late pregnancy). Neonatal infection can be fatal and may be of early onset (occurring within the first 24 hours to first week of life) or late onset (7 days to 6 weeks after birth) and is usually nosocomially acquired. Early onset disease (EOD) accounts for 62% of cases and may manifest as sepsis, respiratory distress and thrombocytopenia, while late onset disease (LOD) may present as sepsis or meningitis. Case fatalities in neonates are high [4] (Okike et al., 2013).

The 2017/2018 outbreak of listeriosis in South Africa, highlighted the need for adequate surveillance and prevention of this infection, bearing in mind that prior to this outbreak, the NICD regarded *Listeria monocytogenes* as a rare pathogen in meningitis infection in South Africa (NICD 2017) [5].

Mathematical modeling has been utilised in the fight against various epidemics. In particular, there is a vast literature on so-called compartmental models of systems of ordinary differential equations (ODEs), [6–9] for instance. Such models are mainly utilized under the assumption that there are no external forces working on the system. Understanding exactly how a sudden epidemic outbreak occurs will often require a different analysis. Such analyses of outbreaks have been studied for various diseases, [10], [11] and [12].

The aim of this paper is to propose a compartmental model for the population dynamics of listeriosis, and to derive a model for analysing a listeriosis outbreak. In particular the model explicitly accommodates neonatal infections. Similarly as is commonly done for cholera models, for instance [7–9], we include a compartment to represent the reservoir of bacteria. We also include a compartment to represent the incubation phase. For the 2017/18 outbreak [13] that happened in South Africa, we calculate the time pattern and intensity of the force of infection, and we make projections on the numbers of new infections and mortalities subsequent to the recall of the contaminated food that was the source of the infection. The model is calibrated using data from the NICD [13] of South Africa, together with existing data in the open literature not necessarily from South Africa. Our model differs from both the listeriosis population dynamics model in [14] and the listeriosis-anthrax coinfection model in [6] in several ways. Essentially the latter two models do not explicitly consider neo-natal infections and do not include a compartment for the incubation phase.

There are different ordinary differential equations (ODE) models for the population dynamics of infectious diseases under different conditions. Usually such models assume that the infection has entered the population or the system, and thereafter there is no external interference on the system. We shall refer to such models simply as population models. Modelling of how the population responds (numerically) while the infection is invading the system, i.e., during an outbreak of the disease, requires a different approach. In this paper we propose a population model for listeriosis disease. Properly calibrated, the model permits computation of future outlooks on a listeriosis epidemic. In terms of this proposed model we reflect on the listeriosis outbreak in South Africa 2017-18 [13]. In particular we discover the temporal pattern description of how the original infection took place and we make future projections of its epidemiology. For a previous listeriosis outbreak, the paper [12] presents a similar investigation but with completely different methods.

## Methods

The population dynamics of listeriosis disease is studied by way of a newly introduced compartmental model of ODE. The model explicitly accommodates neonatal infections and we include a compartment to represent the reservoir of bacteria. Recent examples of models with vertical transmission can be found in the papers [15] and [16] for instance. Our analysis includes both theoretical mathematics as well as computer simulations. The model is calibrated to South African data.

### The model

We describe our ODE compartmental model as follows. By E we denote the collection of individuals who are infected with listeriosis, but do not experience the symptoms. Those who are beyond the latter phase and are suffering the symptoms of listeriosis form a compartment I. By R we denote the removed, i.e., those who have recovered from listeriosis and have temporary immunity. This can be bodily immunity or through lifestyle adaptation. The remaining population forms the class S of so-called susceptibles. At any given time *t* the sizes of these compartments are denoted by *E*(*t*), *I*(*t*), *R*(*t*) and *S*(*t*) respectively. The size of the population at time *t* is denoted by *N*(*t*), and then *N*(*t*) = *S*(*t*) + *E*(*t*) + *I*(*t*) + *R*(*t*). It is also assumed that the pathogen is present in the environment, over and above those that live in the form of human infection. Thus our system has another compartment B, with *B*(*t*) representing the abundance of the pathogen at time *t*. The data in the report [13] of the NICD show that in any given week the number of new infections were always below 1000, in a very large population. This has implications (mainly simplification) of our computations. Since we do not have data available to feed into the model, we work with *B*(*t*) just as an abstract quantity, but we now declare the (proportional) connection of *B*(*t*) with the concentration of the pathogen. If we write *P*(*t*) for the average (over the population) concentration of the pathogen in the diet, then for a suitable proportionality constant *q*, we write *B*(*t*) = *qP*(*t*). Here *q* is chosen in such a way that:

$$\begin{array}{l} B=1\;corresponds\;to\;the\;value\;of\;P\;that,\;in\;a\;population\;of\;60\;million\;susceptibles,\;causes100\;\text{inf}ections\;per\;week. \end{array}$$ (1)

The sizes of these compartments will be treated as continuous variables (rather than integers). We assume a homogeneously mixing population. We introduce non-negative constant parameters *K*, *μ*, *ϕ*, *ψ*, *δ*, *ζ* and *ν*. The number *K* is the size of the population when in disease-free state, i.e., at disease-free equilibrium. The general mortality rate is denoted by *μ*. In the I-compartment the mortality rate is amplified by the disease-induced mortality rate *δ*. Therefore in the compartments S,E,R and I respectively, the rate of outflow due to death are (resp.) *μS*, *μE*, *μR* and (*μ* + *δ*)*I*.

It is assumed that there is an inflow of individuals (newborns) into the system at a rate *μK*. Part of the inflow is into the I-class, as neonates are at risk of contracting the infection from the mother if she is infected. So let us use *θ* to denote the fraction of neonates from infected mothers who do contract the infection from the mother. The number of babies from infected mothers can be calculated as *μI*. Therefore the number of newborns entering the I-class is *θμI*.

**Fig 1:**
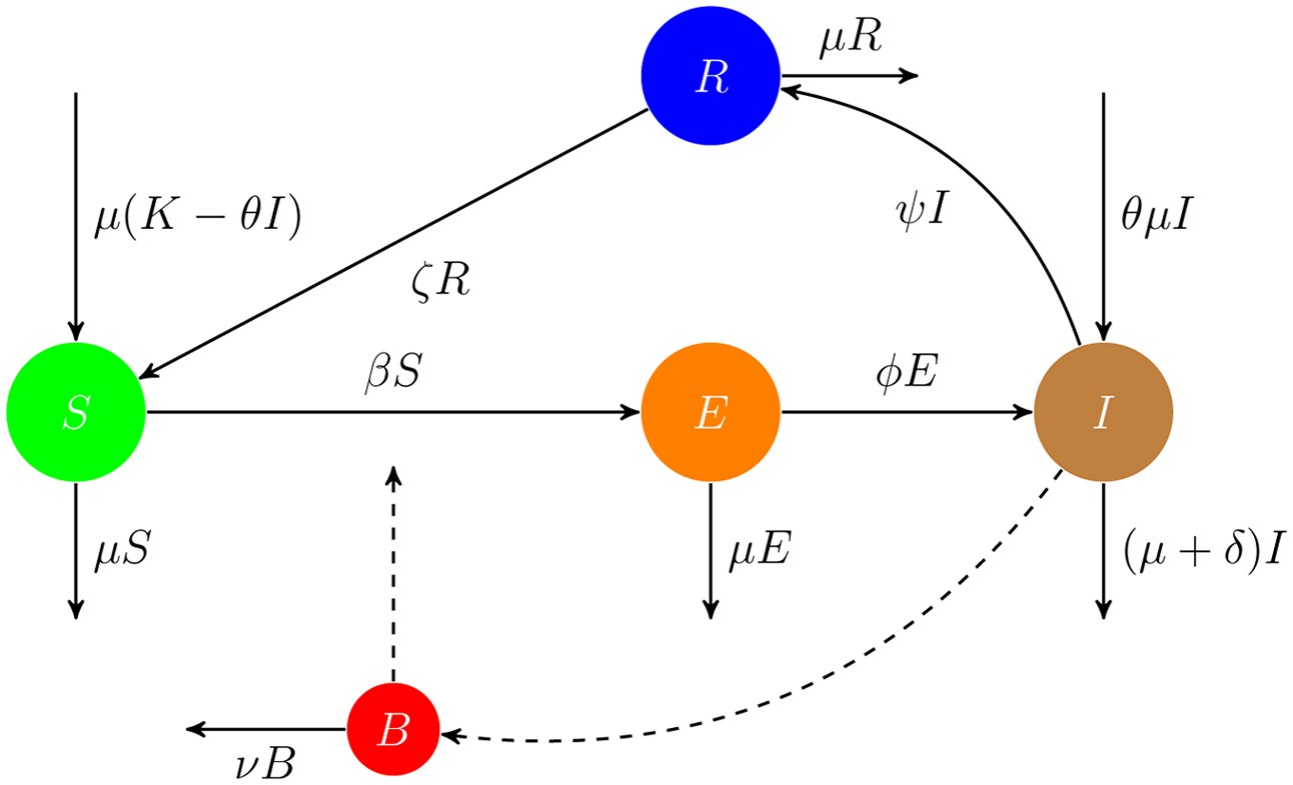
The *flow chart* of the listeriosis disease model. The dashed lines indicate influences but not flow of individuals.

We assume that the probability of an individual becoming infected with listeriosis during a short period of time [*t*, *t* + *h*] is *hβ*(*t*) where *β*(*t*) is given by

$$\begin{array}{c} \beta \left(t\right)=\frac{cB(t)}{L+B(t)} \end{array}$$ (2)

for some fixed positive constants *c* and *L*. This means that new infections occur at a rate *β*(*t*)*S*(*t*), and so *β*(*t*)*S*(*t*) is the rate of transfer from the S-class to the E-class. The given form of *β*(*t*) is also applied in modeling of cholera. The rate of transfer from the E-class to the I-class is *ϕE*(*t*) and the rate of transfer from the I-class to the R-class is *ψI*(*t*). From the R-class, individuals move to the S-class at the rate *ζR*(*t*). The flow chart, Fig 1, of the proposed model shows the rates of transfer in or out of compartments.

In the environment, the bacteria are assumed to have an effective rate of decline denoted by *ν*. Furthermore, the infectious class *I* of the human population contributes to the bacteria in the environment at a rate *ξI*(*t*). The factor *ξ* takes the form

$$\begin{array}{c} \xi =\frac{\eta }{1+\eta_{0}I} \end{array}$$

for some non-negative constants *η* and *η*_0_. Then the population dynamics of the disease is described by the system (3) of five ordinary differential equations.

$$\begin{array}{ccc} \frac{dS(t)}{dt} & = & \mu K-\theta \mu I(t)-\mu S(t))-\beta (t)S(t)+\zeta R(t)\\ \frac{dE(t)}{dt} & = & \beta (t)S(t)-(\phi +\mu )E(t)\\ \frac{dI(t)}{dt} & = & \theta \mu I(t)+\phi E(t)-(\psi +\mu +\delta )I(t)\\ \frac{dR(t)}{dt} & = & \psi I(t)-(\zeta +\mu )R(t)\\ \frac{dB(t)}{dt} & = & \xi I(t)-\nu B(t) \end{array}$$ (3)

For notational convenience we introduce the symbol

$$\begin{array}{c} \mu_{1}=\psi +\mu +\delta . \end{array}$$

The state of the population at any time *t* is a solution *X*(*t*) = (*S*(*t*), *E*(*t*), *I*(*t*), *R*(*t*), *B*(*t*)) of this system of differential equations. As is common in compartmental ODE models, this system of equations is applicable when studying the phase subsequent to an outbreak. During the outbreak itself, the infection is imposed on susceptibles by an external force, and we follow a slightly different approach.

It can routinely be shown that the set

$$\begin{array}{c} D=\{x\in {\left[0,\infty \right)}^{5}:\;x_{1}+x_{2}+x_{3}+x_{4}\leq K\} \end{array}$$

is an invariant domain for the system. This means essentially that all the state variables will be non-negative and the total population size will never be bigger than *K*.

### The basic reproduction number

The model has a disease-free equilibrium point, denoted by $X_{0}^{*}$, and $X_{0}^{*}=\left(K,0,0,0,0\right)$. The basic reproduction number *R*_0_ of a disease in a population is defined as the number of infections caused by introducing a single infectious person into a susceptible population. It has the property that whenever *R*_0_ < 1, then the disease-free equilibrium is locally asymptotically stable, see [17]. The latter means that if the initial state of the population is sufficiently close to disease-free, then in the long run the disease will be eradicated from the system. We determine the basic reproduction number of the model (3) following the next-generation matrix method as presented in the work [17]. The vector of infectious classes is (*E*, *I*, *B*). Then the next-generation matrix is of the form *FV*^−1^ with *F* and *V* as below.

$$\begin{array}{c} F=[\begin{array}{ccc} 0 & 0 & \frac{cK}{L}\\ 0 & \theta \mu & 0\\ 0 & \eta & 0 \end{array}] \end{array}$$

$$\begin{array}{c} V=[\begin{array}{ccc} \phi +\mu & 0 & 0\\ -\phi & \mu_{1} & 0\\ 0 & 0 & \nu \end{array}]. \end{array}$$

Therefore we obtain the next-generation matrix as:

$$\begin{array}{c} FV^{-1}=[\begin{array}{ccc} 0 & 0 & \frac{cK}{\nu L}\\ \frac{\theta \mu \phi }{\mu_{1}\left(\mu +\phi \right)} & \frac{\theta \mu }{\mu_{1}} & 0\\ \frac{\eta \phi }{\mu_{1}\left(\mu +\phi \right)} & \frac{\eta }{\mu_{1}} & 0 \end{array}]. \end{array}$$

Its characteristic equation is:

$${\left(-\lambda \right)}^{2}[\frac{\theta \mu }{\mu_{1}}-\lambda ]+\frac{cK}{\nu L}[\frac{\eta \phi }{\mu_{1}(\mu +\phi )}]\lambda =0.$$

The basic reproduction number is the maximum of the moduli of the roots *λ* of the latter cubic equation. Therefore,

$$\begin{array}{c} R_{0}=\frac{\theta \mu }{2\mu_{1}}+\sqrt{{\left(\frac{\theta \mu }{2\mu_{1}}\right)}^{2}+\frac{\eta \phi cK}{\mu_{1}\left(\mu +\phi \right)\nu L}\;.} \end{array}$$

Recall that the number *R*_0_ serves as an indicator of local stability (or otherwise) of the disease-free equilibrium of system (3) (see [17]). We shall also refer to the following number *R_g_*, when studying global stability, proving that the condition *R_g_* < 1 guarantees global stability of the disease-free equilibrium. Global stability of the disease-free equilibrium means that irrespective of the initial state of the population, in the long run the disease will tend towards extinction, and is a stronger condition than local stability.

$$\begin{array}{c} R_{g}=\frac{\phi \eta cK}{\left(\mu +\phi \right)[\psi +\mu (1-\theta )+\delta ]\nu L}\;. \end{array}$$

In fact, as indicators of stability, the invariants *R*_*g*_ and *R*_0_ are equivalent as we prove below.

**Theorem**. (Equivalence of stability thresholds).

1. *If* *R*_*g*_ < 1 then *R*_0_ < 1.
2. *If* *R*_*g*_ > 1 then *R*_0_ > 1.

**Proof**. We can write

$$\begin{array}{c} R_{g}=\frac{D}{\mu_{1}\left(1-2x\right)},\;\;\text{with}\;\;D=\frac{\phi \eta cK}{(\mu +\phi )\nu L}\;\;\text{and}\;\;x=\frac{\mu \theta }{2\mu_{1}}\;. \end{array}$$

Then

$$\begin{array}{c} R_{0}=x+\sqrt{x^{2}+D/\mu_{1}}\;. \end{array}$$

(a) Now suppose that *R*_*g*_ ≤ 1. Then *D*/*μ*_1_ < (1 − 2*x*). Therefore,

$$\begin{array}{c} R_{0}=x+\sqrt{x^{2}+D/\mu_{1}}<x+\sqrt{x^{2}+1-2x}=x+\sqrt{{\left(1-x\right)}^{2}}=1. \end{array}$$

This proves (a).

(b) This follows by a similar argument.

### Global stability of the disease-free equilibrium

When working towards eradication of an endemic infectious disease, global stabilization of the disease-free equilibrium state is important. This will ensure that in the long term, the pathogen will become extinct in the population.

#### Global stability theorem

*If R*_0_ < 1, then the disease free equilibrium, X* = (*K*, 0, 0, 0, 0), *of the system* (1) *is globally asymptotically stable*.

**Proof**. Let us assume that *R*_0_ < 1. Then also *R*_*g*_ < 1. This condition is equivalent to the inequality

$$\begin{array}{c} \frac{\phi cK}{(\phi +\mu )L}-[\psi +\mu (1-\theta )+\delta ]\frac{\nu }{\eta }<0. \end{array}$$

Given the latter inequality there exist positive numbers *b*_0_, *b*_1_, *ϵ* such that

$$\begin{array}{c} {}[b_{1}+\left(b_{0}+1\right)\frac{\phi }{\phi +\mu }]\frac{cK}{L}-[\psi +\mu (1-\theta )+\delta ]\frac{\nu }{\eta (1+\epsilon )}<0. \end{array}$$ (4)

We fix such numbers *b*_0_, *b*_1_, and *ϵ*. Now we define the following numbers:

$$\begin{array}{c} a_{1}=\frac{(b_{0}+1)\phi }{\phi +\mu }\;\;\text{and}\;\;a_{3}=\frac{\psi +\mu (1-\theta )+\delta }{\eta (1+\epsilon )}. \end{array}$$

In particular we note that

$$\begin{array}{c} \theta \mu -\left(\psi +\mu +\delta \right)+a_{3}\eta <0. \end{array}$$

Therefore we can find a number *a*_0_ > 0 such that *a*_0_ ≤ *b*_1_ and such that

$$\begin{array}{c} \left(a_{0}+1\right)\theta \mu -\left(\psi +\mu +\delta \right)+a_{3}\eta <0. \end{array}$$

We can also choose a number *a*_2_ > 0 such that

$$\begin{array}{c} \left(a_{0}+1\right)\theta \mu -\left(\psi +\mu +\delta \right)+a_{2}\psi +a_{3}\eta <0. \end{array}$$ (5)

We introduce a new variable *Q*(*t*) = *K* − *S*(*t*) and we define a function

$$\begin{array}{c} V=V(Q(t),E(t),I(t),R(t),B(t)), \end{array}$$

which we shall prove to be a Lyapunov function with respect to the disease-free equilibrium point $X_{0}^{*}$. This will suffice to complete the proof. Thus, let

$$V=a_{0}Q+a_{1}E+I+a_{2}R+a_{3}B.$$

Then

$$\begin{array}{c} \frac{dV}{dt}=-a_{0}\mu Q+\left(a_{0}+a_{1}\right)\beta S+E\left[\phi -a_{1}\left(\phi +\mu \right)\right]+I\left[a_{0}\theta \mu +\theta \mu -\left(\psi +\mu +\delta \right)+a_{2}\psi +a_{3}\xi \right]+R\left[-a_{2}\left(\zeta +\mu \right)-a_{0}\zeta \right]-a_{3}\nu B. \end{array}$$

Noting that *β* = *cB*/(*L* + *B*) and *S* ≤ *K*, in particular we obtain the following inequality

$$\begin{array}{c} (a_{0}+a_{1})\beta S-a_{3}\nu B\leq \left[\left(a_{0}+a_{1}\right)\frac{cK}{L}-a_{3}\nu \right]B. \end{array}$$

Also, *ξ* ≤ *η*. Therefore we obtain:

$$\begin{array}{c} \dot{V}\leq -a_{0}\mu Q+E\left[-b_{0}\phi \right]+I\left[a_{0}\theta \mu +\theta \mu -\left(\psi +\mu +\delta \right)+a_{2}\psi +a_{3}\eta \right]+R\left[-a_{2}\left(\zeta +\mu \right)-a_{0}\zeta \right]+B\left[\left(a_{0}+a_{1}\right)\frac{cK}{L}-a_{3}\nu \right]. \end{array}$$

On the right hand side of the latter inequality, we note that the coefficients of *Q*, *E* and *R* are negative. From the inequality (5) it follows that also the coefficient of *I* is negative. Regarding the coefficient of *B* we observe:

$$\begin{array}{c} (a_{0}+a_{1})\frac{cK}{L}-a_{3}\nu =[a_{0}+\frac{(b_{0}+1)\phi }{\phi +\mu }]\frac{cK}{L}-a_{3}\nu . \end{array}$$

Therefore

$$\begin{array}{c} (a_{0}+a_{1})\frac{cK}{L}-a_{3}\nu \leq [b_{1}+\frac{(b_{0}+1)\phi }{\phi +\mu }]\frac{cK}{L}-[\frac{\psi +\mu (1-\theta )+\delta }{\eta (1+\epsilon )}]\nu , \end{array}$$

and so by the inequality (4) the coefficient of *B* is negative. Thus we have shown that *V* is a Lyapunov function, and this completes the proof.

### Existence of an endemic equilibrium point

The endemic equilibrium point $X_{e}^{*}$ has I* ≠ 0. In this case, in particular we have also B* ≠ 0. In fact for the endemic equilibrium point we have (and note that we write *η*/(1 + *η*_0_ I*) = ξ*):

$$\begin{array}{c} I^{*}=\frac{\nu }{\xi^{*}}B^{*},\;R^{*}=\frac{\psi }{\zeta +\mu }I^{*},\;E^{*}=\frac{m\nu }{\phi \xi^{*}}B^{*}, \end{array}$$

where *m* = *ψ* + *μ* + *δ* − *θμ*. The values of S* and B* are calculated as follows:

$$\begin{array}{c} S^{*}=\frac{(\phi +\mu )m\nu }{c\phi \xi^{*}}\left(L+B^{*}\right), \end{array}$$

$$\begin{array}{c} B^{*}=\frac{\mu (\zeta +\mu )}{\nu Z}[c\phi \xi^{*}K-\left(\phi +\mu \right)m\nu L], \end{array}$$ (6)

$$\begin{array}{c} \text{with}\;\;Z=c\phi \theta \mu (\zeta +\mu )+m(\zeta +\mu )(\phi +\mu )(\mu +c)-c\zeta \psi \phi . \end{array}$$

Now we note the following inequality:

Z>m(ζ+μ)(ϕ+μ)(μ+c)−cζψϕ>m(ζ)(ϕ)(c)−cζψϕ>0,becausem>ψ.

Thus we have shown that the denominator in the expression (6) is positive. Consequently for B* > 0 we must have cϕξ*K − (*ϕ* + *μ*)*mνL* > 0. The latter condition implies *R*_0_ > 1, since ξ* ≤ *η*. It can routinely be shown that the positive equilibrum $X_{e}^{*}$ exists if and only if *R*_0_ > 1.

### Parameter values relevant to outbreak

Values of parameters of system (3) will be discussed in this section, and are presented in Table 1. We also analyse the transmission probabilities *α*(*t*) for the duration of the outbreak. Since the listeriosis data [13] from the South African NICD are given per week, we choose 1 week as our unit of time (but in simulations we consistently use timestep equal to 1 day).

**Table 1:** Numerical values of parameters.

Param.	Description	Numerical value	Reference/comment
*K*	Population size when disease-free	60 000 000	[18]
*μ*	mortality rate, excluding death directly due to listeriosis	0.000298 per week	[18]
*δ*	rate of human deaths due to listeriosis	0.0183 per week	[13]
*ϕ*	Transfer rate from E-class to I-class	1.29 per week	[13]
*ψ*	Transfer rate from I-class to R-class (recovery rate)	0.0484 per week	[13]
*ζ*	Transfer rate from R-class to S-class (rate of loss of becoming susceptible post recovery)	$\frac{1}{6}$ per week	Nominal value, lack of available data. This depends on the specific population/region.
*θ*	The proportion of neonates who contracts listeriosis from the mother	0.9	[13]
*c*	a proportionality constant	3.33 × 10^−6^ per week	See the description of *B*
*L*	A threshold value of *B*	1	Nominal, lack of data
*ν*	Removal rate of pathogen from the environment	2 units per week	Nominal value, lack of available data. This depends on the specific population/region.
*η*	associated with the effect of infectious humans on the bacterial reservoir	0.00137 per week	estimated (led by [19])
*η*_0_	associated with the effect of infectious humans on the bacterial reservoir	1/71.6 per person	nominal

From the NICD data which are reflected in Fig 3, we fit the parameters *ϕ*, *ψ* and *δ*, and their values are as in Table 1. The data also sheds light on *θ*. Regarding *θ*, simple calculations reveal that there must have been many infected babies whose mothers were not listeriosis patients. In a random sample of *n* the expected percentage *F* of babies, who in a given period of 62 weeks were less than 28 days old, is calculated as

$$\begin{array}{c} F=[\mu \times (62+4)\times n]/n=1.97.\% \end{array}$$

The percentage of neonates among the infected is reported in [13] as 42% which is bigger by a factor of more than 20. This leads one to conclude that if both a mother and her neonate was exposed to *L. monocytogenes*, then the baby was more likely to contract the disease than the mother. In particular then, for the given scenario one would suspect that if the mother of a neonate was infected, then her child would very likely also be infected. On this basis we guess a value, *θ* = 0.9. The other parameters are obtained from the literature (except for *L*, *η* and *ν*, which are not relevant to the outbreak).

In the census document [18] the average life expectancy in South Africa is given as 61.2 years for males and 66.7 years for females. Therefore we calculate the value of the general mortality rate *μ* for South Africa as $\frac{1}{\left(64.45)(52\right)}=0.000298$ per week.

Members in the class R are considered at least temporarily safe from becoming infected, due to bodily immunity or through lifestyle adaptation. In the case of listeriosis there are clear guidelines for avoiding infection, generally published regularly by public health authorities, see [13] or [20] for instance. The effective duration of the temporary immunity is taken as 6 weeks, and so *ζ* = 1/6 per week.

Regarding the function *β*, it is difficult to find an optimal value for *L*. However, we assign to it a nominal value *L* = 1. Then the value of *c* is fixed by the definition of *B*(*t*): Since *B* = 1 corresponds to 100 new latent infections per week and we have chosen *L* = 1, we obtain *c* × 1 × 6 × 10^7^/(1 + 1) = 100. This means that *c* = 1/300000 (per week).

The parameters *η*, *η*_0_ and *ν* will be given nominal values. They are not relevant to the outbreak and we shall explain them at a later stage.

**Remark** (a) A value for *ϕ* can be estimated based on the papers [21] and [22]. The incubation period of listeriosis varies quite significantly: from under 24 hours up to as much as 90 days—a study of the incubation period appears in [21] for instance. A final value is not concluded in [21]. Our fitted value of *ϕ*, puts the average incubation period for listeriosis at just below 5.5 days.

(b) In the study [22] it is observed that the mortality among the infected is one-third. The period post incubation, subsequent to the pathogen having taken effect, over which these deaths occur was not specified. We note that treatment for listeriosis takes 2-6 weeks (Bacteremia should be treated for 2 weeks, meningitis for 3 weeks, and brain abscess for at least 6 weeks, [20]). So if one takes the duration of the infectious period as 6 weeks, then it suggests a disease-induced mortality rate of 1/(3 × 6) per week. This is approximately three times the rate that we calculate for this outbreak.

## Results

### During the outbreak

In what follows we present a numerical analysis of the force of infection, *α*(*t*), during the outbreak period, 01 January 2017 up to 08 March 2018. We compare the model output of the mortalities and recoveries with the numbers reported in [13], and we present an outlook on the return of the system to equilibrium subsequently to the recall of the food products that caused the outbreak.

As noted earlier, the original system of equations is applicable when studying the phase subsequent to the outbreak, when there are no external forces on the system. During the outbreak the infection is imposed on susceptibles by an external force. This force is assumed to be resulting in infections at a rate *α*(*t*)*S*(*t*), where *α*(*t*) is a function of time. This *α*(*t*) replaces *β*(*t*) as the driving force of the infection, it does not have the same functional form as the *β*(*t*) of the population model and is unknown. In order to assess the force of infection, *α*(*t*), during the outbreak period itself, we shall utilise the system (7) below. System (7) is obtained from system (3) by excluding the fifth differential equations and the force of infection will be determined from the data [13]. Then we have a system (7) of four ordinary differential equations.

$$\begin{array}{ccc} \frac{dS(t)}{dt} & = & \mu K-\theta \mu I(t)-\mu S(t))-\alpha (t)S(t)+\zeta R(t)\\ \frac{dE(t)}{dt} & = & \alpha (t)S(t)-(\phi +\mu )E(t)\\ \frac{dI(t)}{dt} & = & \theta \mu I(t)+\phi E(t)-(\psi +\mu +\delta )I(t)\\ \frac{dR(t)}{dt} & = & \psi I(t)-(\zeta +\mu )R(t) \end{array}$$ (7)

The system (7) above is discretized as follows, with *h* being the time increment.

$$\begin{array}{ccc} S(t+1) & = & S(t)+(\mu K-\theta \mu I(t)-\mu S(t))-\beta (t)S(t)+\zeta R(t))h\\ E(t+1) & = & E(t)+(\alpha (t)S(t)-(\phi +\mu )E(t))h\\ I(t+1) & = & I(t)+(\theta \mu I(t)+\phi E(t)-(\psi +\mu +\delta )I(t))h\\ R(t+1) & = & R(t)+(\psi I(t)-(\zeta +\mu )R(t))h \end{array}$$ (8)

Initial values for the compartmental variables are chosen as below, which we shall see eventually are the endemic equilibrium values corresponding to the parametrization given in Table 1:

E(0)=4,I(0)=72,R(0)=21

**Fig 2:**
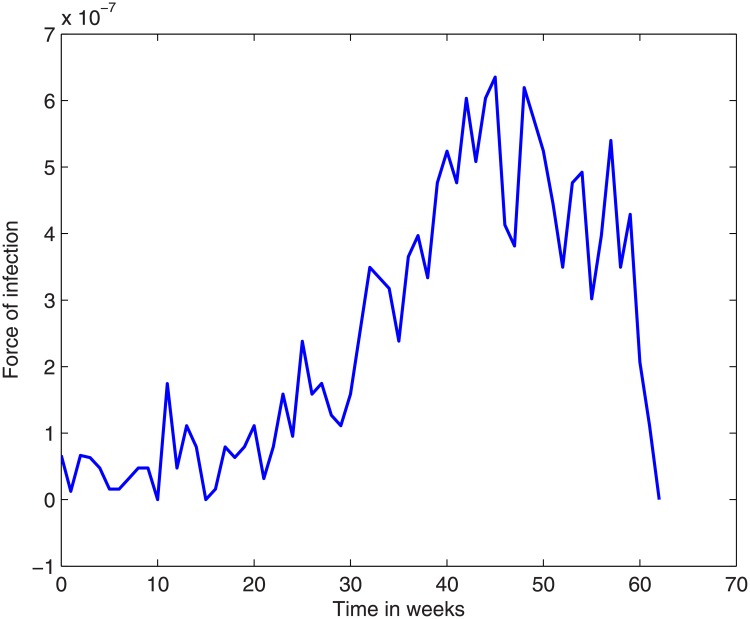
The infection shock *α*(*t*) as calculated from the data over the outbreak period, 01 January 2017 up to 08 March 2018.

The new cases for each week can be read from the data in [13]. During the *t*’th week this number is the same as (*ϕE*(*t*) + *θμI*(*t*)), and the expression for *E*(*t*) contains the variable *α*. These can be used to calculate the function *α*(*t*), which we display in Fig 2.

**Fig 3:**
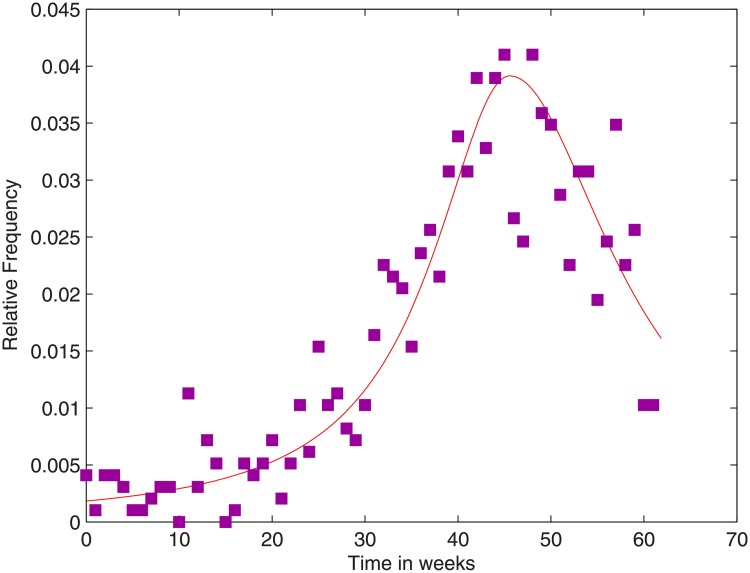
Scatter diagram of the relative number of cases over the outbreak period and the density plot.

In order to smooth out the NICD data, we found a density plot, *p*(*t*), for the relative number of cases, i.e., (cases)/967. This density plot is shown together with the scatter diagram of relative case numbers in Fig 3. This *p*(*t*) will be used hereafter to describe the force of infection over the outbreak period, in a similar way as is done for the rough data.

### Comparing the model output with reported results

In Table 2 we present three outputs of the model for comparison with reported results [13], for the period 01 January 2017 up to 08 March 2018. We give the total new cases as reproduced on the basis of the force of infection, computed using the Eq (8) above. The three outputs that we compute are the total number of cases, the total number of fatalities and the total number of recoveries. We are then able to calculate the number of remaining infectives through simple arithmetic. The outputs are calculated twice: i.e., first for rough data and then from the density plot. In both cases the outcomes compare very well with the observed data.

**Table 2:** The model output versus observed data.

Total number of individuals who …	NICD Report [13]	Model output rough data	Model output density plot
became infected during the 62 weeks period	967	967.5	964.3
died during the 62 weeks period	183	183.3	182.7
was infected and has recovered during the 62 weeks period	486	487.6	484.7
are infected on 08 March 2018	298	296.6	296.7

**Fig 4:**
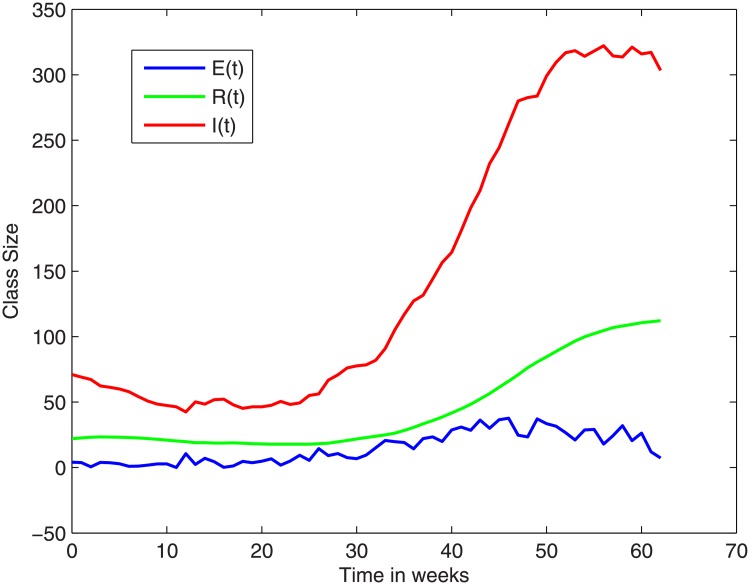
The trajectories of the E, I and R -classes over the outbreak period, based on the rough data.

**Fig 5:**
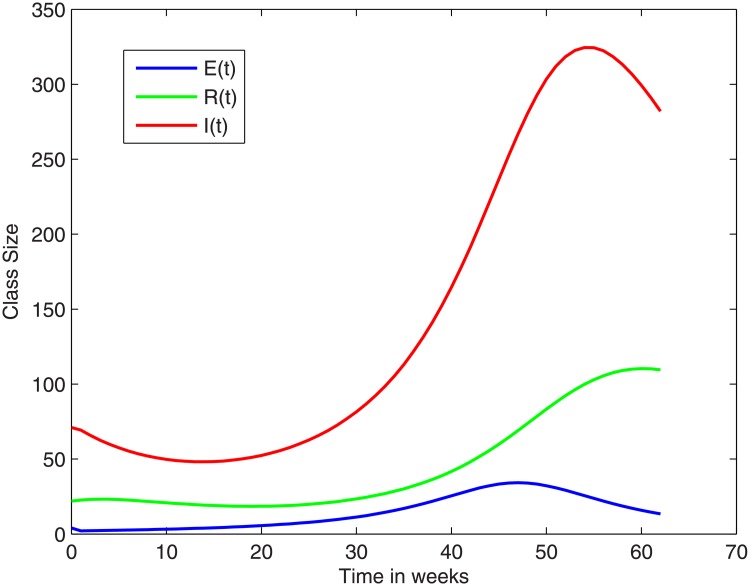
Trajectories of the E, I and R -classes over the outbreak period, using the density plot.

In Fig 4 (based on the NICD-data) and Fig 5 (based on the density plot) we show the trajectories of the E-, I-, and R-compartments over the outbreak period. The S-class is many orders bigger. In fact, we note that the total number of cases during the outbreak was fewer than 1000. Therefore if we consider *S*(0) to be 60 million, then during the outbreak *S*(*t*) will always be above 59999000.

### The complete population model

What remains to be determined are the parameters of the last (the pathogen’s) equation. South Africa’s Minister of Health in his media report of 03 September 2019 [19] announced that NICD records show between 60 and 80 mortalities p.a. due to listeriosis, over the years 2012-2016. Let us take this as approximately 70 listeriosis deaths p.a. under equilibrium conditions. This enables us to calculate I* as follows:

δI* × 52 = 70, therefore I* = 71.6.

We note that at endemic equilibrium, from the second and the third equations of the system (3) we have:

$$\begin{array}{c} \frac{cB^{*}S^{*}}{L+B^{*}}=\left(\phi +\mu \right)E^{*}\;\;\text{and}\;\;\phi E^{*}=mI^{*}. \end{array}$$

This yields

$$\begin{array}{c} B^{*}=\frac{m\left(1+\mu /\phi \right)I^{*}}{cS^{*}-m\left(1+\mu /\phi \right)I^{*}},\;\;\text{so}\;\;B^{*}=0.0245\;. \end{array}$$

Therefore we have calculated the value of B* in terms of the known parameters, the values of which are well motivated. In particular we did not require *ν*, *η* or *η*_0_. Thus we are in a position to calculate the ratio ξ*/*ν* = B*/I*. This yields ξ*/*ν* = 0.000342. So, in order to complete the calibration of the model, we only need to determine the value of either ξ* or *ν*. For both of the latter two parameters, their values depend on the environment together with human behaviour and lifestyle. So for any two different populations, there may be a huge difference between the two *η* -values. Likewise for *ν* -values. We pick a nominal value *ν* = 2 units per week. Then we use *η*_0_ = 1/71.6 and that automatically determines *η*:

$$\begin{array}{c} \eta =\left(1+\eta_{0}I^{*}\right)\xi^{*}=\left(1+\eta_{0}I^{*}\right)0.000353\nu =2\times 0.000342\times 2=0.00137\;. \end{array}$$

**Fig 6:**
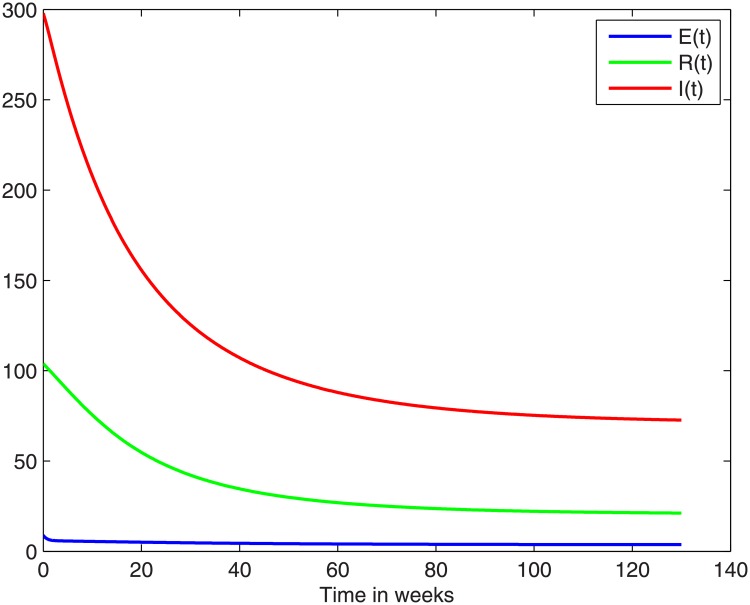
The return to endemic stable state over a period of 130 weeks, 08 march 2018–30 june 2020, according to the model. See also Table 3.

Now we can harness the model for making future projections. The graphs in Fig 6 illustrate how the population returns to equilibrium subsequent to removal of the cause of the outbreak. Of course the initial values of this computation are the terminal value of the outbreak computation. In particular, the initial value of *B*(*t*) is taken as the value of *B*(*t*) that was built up during the outbreak, i.e., *B*(end of outbreak). During the outbreak period *B*(*t*) starts at B* = 0.0245 and reaches a final value 0.0409 at the end of the outbreak. Thus for the simulations of Fig 6 we use *B*(0)= 0.0409.

Table 3 shows the equilibrium values as calculated using the formulae and parameter values, and also the values of the variables S, E and I as predicted by the model for the dates 30 June 2019 and 30 June 2020. The prediction values do appear to be converging to the endemic equilibrium.

**Table 3:** Model prediction of listeriosis prevalence in South Africa.

Disease class *X*	X*-value	Model prediction 30 June 2019	Model prediction 30 June 2020
E	3.7	4.0	3.8
I	71.6	80	74
R	21	24.4	21.5
B	0.0245	0.0259	0.0249

### Reducing the annual mortalities

The stability threshold is computed as having the numerical value *R*_0_ = 1.43. The Global Stability Theorem revealed that when *R*_0_ < 1, then in the long run, the disease will vanish from the system. This informs how much should be done by way of intervention to ensure that the disease will not persist and flourish. Interventions should lead to reduction of *R*_0_ below unity.

**Fig 7:**
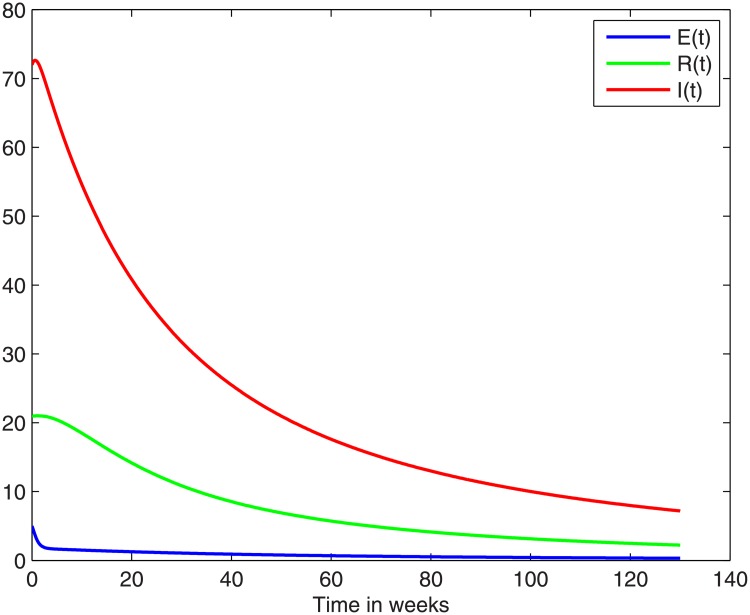
With a change in the value of *η* to *η* = 0.00065, we obtain *R*_0_ = 0.987 which is below 1, and as guaranteed by the Global Stability Theorem, over time the infection will be eradicated. The graph shows how the system will change over a period of 130 weeks, starting from what is currently the endemic equilibrium. We note a definitive tendency of convergence to disease-free state in the longer term.

In particular, with an intervention which changes the parameter values in such a way as to result in *R*_0_ going below unity, the disease will ultimately vanish from the population. In this regard we could immediately consider parameters that depend on behaviour or lifestyle, such as *η*: perhaps through improved sanitation service, or *c*: good hygiene around food and drinking water. Indeed the graph in Fig 7 illustrates, how the infectious class diminishes converging to zero, under a suitable intervention. In this case, the parameter values are all the same as in Table 1, except for *η* which has been reduced to 0.00065 (instead of 0.0014), and this gives *R*_0_ = 0.987 which is below 1.

## Discussion and conclusion

We have constructed a mathematical model for the population dynamics of listeriosis infection. Such a model is useful to public health authorities when exploring intervention strategies to control or eradicate an infectious disease (Huppert and Katriel 2013 [23], Althaus et al 2014 [24]; Rose et al 2014 [25], Heesterbeek et al 2015 [26]). In particular, interventions should aim at reducing the value of the basic reproduction number, bringing it to below unity where possible. In the case of the current model, having *R*_0_ < 1 will guarantee eradication of the disease in the long run. For some disease models, the said condition is not sufficient to ensure eradication, but we proved that for the current model it is indeed.

We were able to harness the model in a modified form, retrospectively to reveal the intensity, or at least the temporal pattern, of the infection which caused the outbreak. The data available is limited, and consequently not all of the parameters could be determined. Nevertheless we were able to obtain a reasonable estimate of the basic reproduction number and the endemic equilibrium point. The endemic equilibrium as calculated yields an endemic equilibrium value of I* which is in line with the annual infection numbers in South Africa (see [19]). We also demonstrated how to compute future projections (Table 3), and how to test an intervention for the purpose of eradication (Fig 7).

For application to another population, the model can be used in the same form, but the parameter values must be adapted. For this reason adequate surveillance systems and resources to diagnose or detect listeria must be in place. Accuracy can be improved by allowing for age structure and spatiality, and to produce simulations through more accurate methods. Climate variability may directly or indirectly lead to higher infection rates of listeriosis in South Africa [27]. Modeling that accommodates the effect of climatic factors, such as was done for malaria by several authors ([28] or [29] for instance) may prove useful in the near future.
